# Intraoperative frozen section can be reduced in thyroid nodules classified as Bethesda categories V and VI

**DOI:** 10.1038/s41598-017-05459-x

**Published:** 2017-07-12

**Authors:** Jing Huang, Jieli Luo, Jianshe Chen, Yang Sun, Chao Zhang, Kanlun Xu, Qin Ye, Pintong Huang

**Affiliations:** 1 Department of Ultrasound, the Second Affiliated Hospital of Zhejiang University School of Medicine, Zhejiang, 310009 China; 2 Department of Pathology, the Second Affiliated Hospital of Zhejiang University School of Medicine, Zhejiang, 310009 China

## Abstract

Intraoperative frozen section (FS) can be reduced during thyroid lobectomy according to the results of fine needle aspiration (FNA). We evaluated the role of intraoperative FS in thyroid nodules with different diagnostic categories of the Bethesda System for Reporting Thyroid Cytopathology by FNA. This retrospective study included 1,235 nodules collected via thyroidectomy with both preoperative FNA and intraoperative FS at the Second Affiliated Hospital of Zhejiang University School of Medicine, from January 2011 to January 2014. FNA cytological diagnosis was classified into six categories, based on the Bethesda system. The diagnostic findings of FNA cytology and FS histology were compared with the final histological results. 189 nodules were benign. The remainder were malignant. FS diagnosis was more accurate than FNA diagnosis for nodules classified as Bethesda Categories II, III, and IV (*P* < *0.05*). However, the accuracy of FNA diagnosis in nodules assigned Bethesda Categories V and VI was significantly higher than that of FS (*P* < *0.05*). FS appears be beneficial for thyroid nodules classified as Bethesda categories I through IV. FS may not be necessary in nodules diagnosed as Bethesda Categories V and VI.

## Introduction

The incidence of thyroid nodules has increased in recent years^1^, with an estimated prevalence of 1–5% for palpable nodules and up to 50% for nonpalpable nodules^2^. This increase in incidence is due to identification through the use of ultrasound examinations and the emphasis on early detection of cancer over the past three decades^3^. Autopsy studies have showed that 50% of the U.S. population has thyroid nodules^4^. The reported incidence of thyroid cancer in all thyroid nodules is approximately 5%^5^.

Ultrasound-guided fine needle aspiration^6^ is the most useful and reliable method for the evaluation of thyroid nodules^7^. It was used worldwide because of its safety, simplicity, and cost-effectiveness^8, 9^. Cytological results are classified according to the Bethesda System for Reporting Thyroid Cytopathology^6^, which has been applied in our institution since 2011. These cytological diagnoses are determined, as follows: 1) Bethesda I, nondiagnostic or unsatisfactory, 2) Bethesda II, benign, 3) Bethesda III, atypical of indeterminate significance or follicular lesion of indeterminate significance, 4) Bethesda IV, follicular neoplasm or suspicious for a follicular neoplasm, 5) Bethesda V, suspicious for malignancy, and 6) Bethesda VI, malignant.

FNA diagnoses may not be definitive. This creates a dilemma with regard to follow-up and/or treatment, especially regarding the need for surgery. Frozen section (FS) has been used extensively to help guide intraoperative management of patients with thyroid nodules. However, FS is usually insufficient to determine true capsular or vascular invasion, and deferral to a final pathologic diagnosis is often necessary in the setting of follicular lesions^10^. The use of intraoperative FS has long been debated, secondary to concerns about increased costs and operative time, without a true consensus^9, 11^. The need for intraoperative FS after a prior conclusive FNA diagnosis is under consideration. The purpose of this study was to retrospectively evaluate the value of FS in assigning different Bethesda categories.

## Results

**Figure 1:**
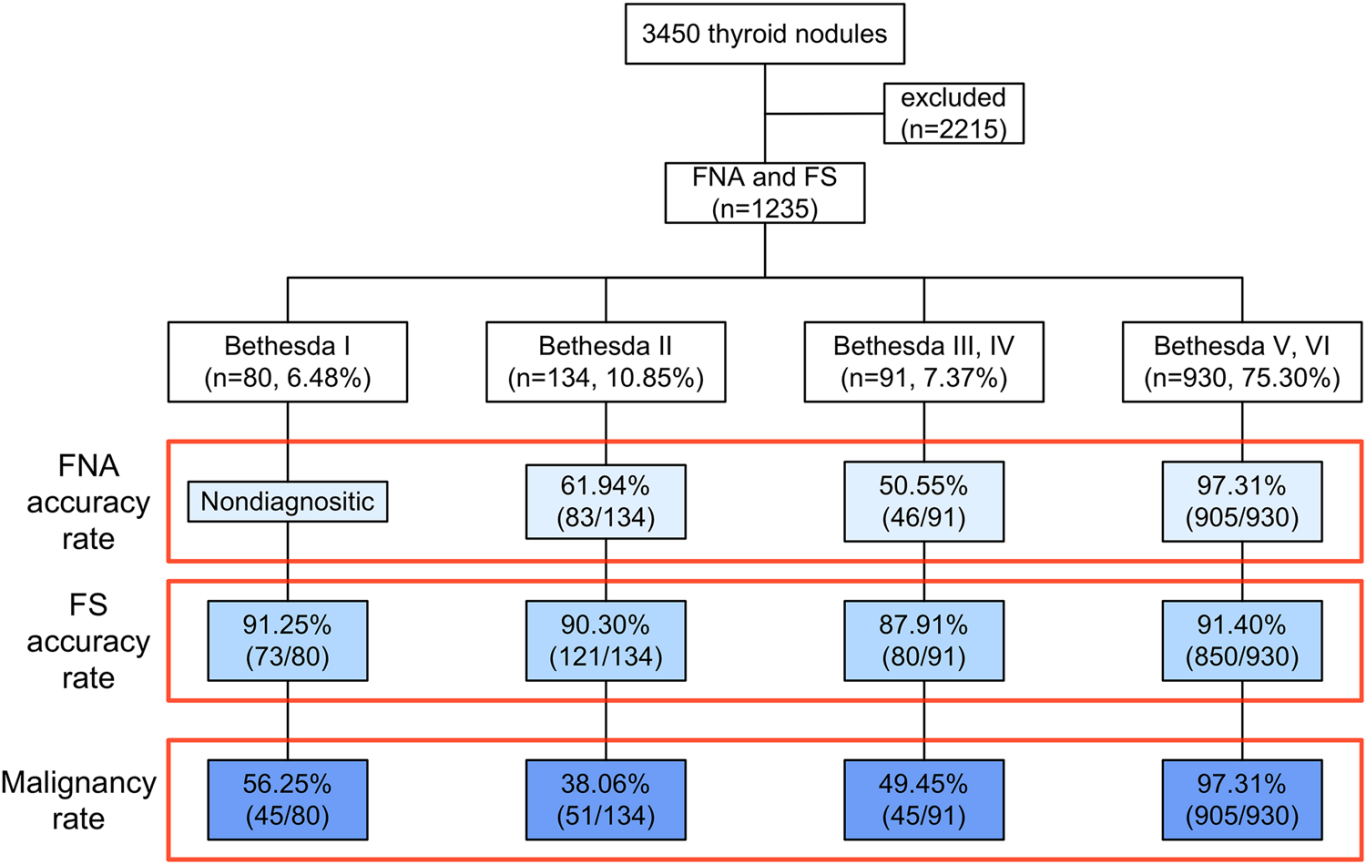
Diagnostic outcomes of 3,450 thyroid nodules. The final diagnoses of all nodules were confirmed by surgical pathology. FNA, fine needle aspiration; FS, frozen section.

1,235 nodules from 1,182 patients were included. Of these nodules, 268 were from males and 967 were from females, a male to female ratio of 1:3.6. The mean age of participants was 46.8 ± 11.7 years, with a range of 13–80 years. 1,046 of the nodules were diagnosed as thyroid malignancies on final histopathology (Fig. 1). Among these malignancies, 1,032 were papillary thyroid carcinomas, 7 were follicular thyroid carcinomas, 4 were Hürthle cell carcinomas, and 3 were medullary carcinomas. Histopathological results of the 189 benign nodules included 90 instances of adenomatous hyperplasia, 82 follicular adenomas, and 17 thyroiditis. A detailed description of the pathological results are shown in Table 1.

**Table 1:** Histological types of 1235 thyroid nodules.

Classification	Pathologic diagnosis	*n* (%)
Malignant		1046 (84.70)
	Papillary thyroid carcinoma	1032 (83.56)
	Follicular thyroid carcinoma	7 (0.57)
	Hürthle cell carcinoma	4 (0.32)
	Medullary carcinoma	3 (0.24)
Benign		189 (15.30%)
	Adenomatous hyperplasia	90 (7.29)
	Follicular adenoma	82 (6.64)
	Thyroiditis	17 (1.38)

Eighty (6.48%) nodules were reported as Bethesda category I (Table 2), of which 56.25% (45/80) were histologically diagnosed as malignant. Seven nodules diagnosed as benign through FS were identified as papillary thyroid carcinomas in the final histologic diagnoses. The accuracy rate of FS was 91.25% in this category. One hundred and thirty-four (10.85%) nodules were classified as Bethesda Category II, of which 38.06% (51/134) were histologically diagnosed as malignant, 38 were reported as malignant on FS, and 13 were false negatives on intraoperative FS, yielding accuracy rates of 90.30% for FS and 61.94% for FNA. The false negative rate for FNA in assigning Bethesda Category II was significantly higher than that of FS (100% vs. 25.49%, *P* < 0.001). There was no significant difference in the negative predictive value between FNA and FS (61.94% vs. 86.46%, *P* > 0.05).

**Table 2:** The ultrasonographic features of Bethesda category I nodules.

Feature	Benign	Malignant	*P* value
Nodule type			0.012
Solid	16	33	
Cystic and solid	19	12	
Echogenicity status			0.000
Hypoechogenecity	10	39	
Hyperechogenecity or isoechoic	25	6	
Nodular edge status			0.915
Irregular	12	21	
Regular	13	24	
Halo			0.000
Without halo	33	10	
With halo	2	35	
Nodular size			0.139
Larger than 10 mm	4	11	
Smaller than 10 mm	31	34	
Calcification			0.001
With calcification	18	38	
Without calcification	17	7	

Ninety-one (7.37%) nodules were classified as Bethesda Categories III or IV. Of these nodules, 49.45% (45/91) were histologically diagnosed as malignant, 34 were reported as malignant on FS, and 11 nodules were false negatives on intraoperative FS, giving accuracy rates of 87.91% for FS and 50.55% for FNA. The false negative rate for FNA in assigning Bethesda Categories III or IV was significantly higher than that of FS (100% vs. 24.44%, *P* < 0.001). The negative predictive value of FNA was also higher than that of FS (50.55% vs. 80.70%, *P* < 0.001).

**Figure 2:**
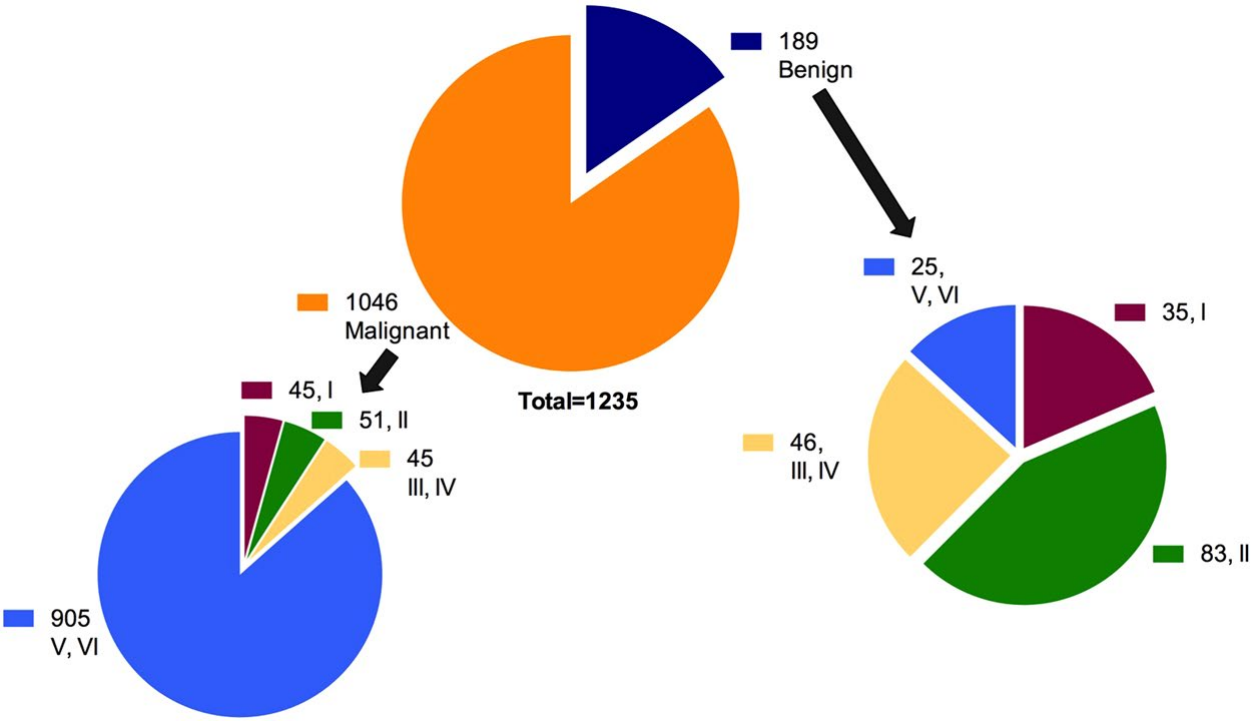
Benign and malignant thyroid nodules based on the Bethesda system. I, Bethesda category I; II, Bethesda category II; III, Bethesda category III; IV, Bethesda category IV; V, Bethesda category V; VI, Bethesda category VI.

Nine hundred and thirty (75.30%) nodules were classified as Bethesda Categories V or VI, of which 97.31% (905/930) were histologically diagnosed as malignant, 825 were reported as malignant on FS, and 80 nodules were false negatives on intraoperative FS. The false positive rate for FNA in Bethesda Categories V or VI was significantly lower than that of FS (0.00% vs. 8.84%, *P* < 0.001), while the positive predictive value of FNA in Bethesda Categories V or VI (97.31%) was significantly lower than that of FS (100%; *P* < 0.001), giving accuracy rates of 91.40% for FS and 97.31% for FNA. Bethesda categories V and VI were most accurately diagnosed via FS, while categories III and IV were least accurately diagnosed. FS diagnosis was more accurate than FNA diagnosis in nodules assigned Bethesda Categories III, III, and IV (*P* < 0.05), but the accuracy of FNA diagnosis in nodules classified as Bethesda Categories V and VI was significantly higher than that of FS (*P* < 0.05; Fig. 2 and Table 3).

**Table 3:** Diagnostic accuracy of FNA and intraoperative FS.

Bethesda Category	FS	Pathology		Accuracy rate		*P* value
		M	B	FNA	FS	
I	M	38	0		91.25%	
	B	7	35			
II	M	38	0	61.94%	90.30%	<0.001
	B	13	83			
III, IV	M	34	0	50.55%	87.91%	0.001
	B	11	46			
V, VI	M	825	0	97.31%	91.40%	<0.001
	B	80	25			

Note: M, malignant; B, benign; FNA, fine needle aspiration; FS, frozen section.

## Discussion

The prevalence of thyroid nodules has been increasing globally during the past three decades because of increased screening; the majority of cases were asymptomatic benign thyroid nodules for which surgical intervention is unnecessary^12^. In contrast to benign nodules, malignant thyroid nodules can lead to lymph node metastasis. Therefore, it is important to evaluate the nature of thyroid nodules^13^. Ultrasound-guided FNA is the method most frequently used to evaluate thyroid nodules with high accuracy, and is less invasive than surgery^9^. However, whether the use of intraoperative FS of thyroid nodules with previous conclusive FNA diagnosis is necessary remains controversial^14, 15^. Richards *et al*. analyzed data from 231 patients and found that conventional FS not only increased the cost of surgery, but also extended the procedure time, with no benefit to the patients^16^. Although intraoperative FS is routinely performed, it has limitations. Thyroid tissue becomes crisp during the fast freezing process, increasing the difficulty of making slices^17^. In addition, changes in nuclear size, architectural distortion, and loss of cellular details occur due to shrinking and dehydration; these changes have a negative influence on the diagnostic accuracy of FS^18^.

For nodules in Bethesda categories V and VI, intraoperative FS results could be replaced with preoperative FNA to select a surgical plan. The most common thyroid malignancy is papillary thyroid carcinoma (PTC)^19^. FNA is the best choice for diagnosing PTC, due to its high sensitivity and specificity in recognizing psammoma bodies and nuclear-specific changes, such as nuclear grooves, pseudo-inclusion bodies, and a ground-glass appearance^20^. Livolsi and Baloch were concerned that FS might not be necessary if the FNA results confirmed PTC^10^. The present study showed that the diagnostic accuracy rate of FNA and FS in identifying nodules classified as Bethesda Categories V and VI were 97.31% (905/930) and 91.40% (850/930), respectively. The false negative rate of FS was 8.84% (80/905), and the accuracy of FNA was higher than that of FS (*P* < *0.05*).

When the thyroid nodules are severely fibrotic, densely calcified, and have hemorrhagic or cystic changes, only inadequate follicular cells are obtained on FNA, which yields cyst content with no cells. In the present study, these nodules were reported as non-diagnostic or unsatisfactory. In this situation, FS was an effective approach and helpful in the management of such patients. Hence, the role of intraoperative FS in assigning nodules to Bethesda category I was investigated here. In Bethesda category I nodules, intraoperative FS demonstrates high levels of accuracy in patients with unsatisfactory FNA (91.25%). The occurrence of malignancy in nodules classified as Bethesda category I was 56.25% (45/80). In these instances, the surgeon could choose the best surgical plan according to intraoperative FS in those patients who continue to receive thyroidectomy after FNA.

Malignancy cannot be excluded, even with a benign FNA result, especially if a nodule has suspicious features on ultrasound^21^. Further FS information from these nodules is required and necessary to confirm the prior FNA diagnosis^22, 23^. Our findings showed that the accuracy rates of FNA and FS were 61.94% (83/134) and 90.30% (121/134), respectively, in Bethesda Category II nodules. Fifty-one (38.06%) malignant nodules in this Category were mistaken for benign nodules by FNA, while the false negative rate for FS was 25.49% (13/51). The reason for the false negative results in FS found here may due to the small size of thyroid cancer in the resection specimen, which may cause difficulty in the gross examination. FS is usually insufficient to determine true capsular or vascular invasion, and deferral to a final pathologic diagnosis is often necessary in the setting of follicular lesions^10^.

In diagnosing nodules with Bethesda categories III and IV, both FNA and FS have limits in predicting the final histological results. Thus, combining these two methods may lead to better treatment outcomes. Our results showed that FS was more accurate than FNA. The diagnosis of malignant follicular tumors depends on features of capsular and vascular invasion, which can only be identified through pathology^10^. LiVolsi and Baloch considered that FS had no value in diagnosing follicular or Hürthle cell tumors^10^. FS might aid in diagnosis when the nodules are highly suspicious of capsular invasion^24^.

This study was limited by grouping the nodules according to the Bethesda system, which could have created in-group bias. In addition, because of the findings from nodules assigned Bethesda category I, the accuracy rate could only be measurable with FS, which may not have captured the nature of those nodules. Furthermore, the cytology determinations were made by a single cytologist. We did not assess the inter- and intra-observer variability in interpretation of cytological results. Finally, nodules classified as Bethesda Categories III and IV were artificially classified as benign nodules, compared malignancy as established through FS.

Given the high accuracy of FNA in Bethesda Categories V and VI, preoperative FNA, rather than intraoperative FS, may be appropriate for those patients. However, it is important to note that in the cases of Bethesda I, II, III, and IV nodules diagnosed using FNA, FS is considered a complementary tool to improve the detection rates and reduce the risks of a second surgery, and should be used together with FNA in those categories.

## Methods

Written informed consent was obtained from all patients before their examinations. The local ethics committee and institutional review board of the Second Affiliated Hospital of Zhejiang University School of Medicine approved this prospective study. The methods in this study were performed in accordance with approved guidelines. No incentives, financial or otherwise, were offered to them.

### Patients

From January 2011 to January 2014, we analyzed 3,450 nodules that underwent US-guided FNA in our Department of Ultrasound. 2,215 nodules were excluded because of lack of clinical data (no follow-up or surgery) (Fig. 1). The remaining 1,235 nodules, which had FNA, FS, and surgery data, were included. Medical records of those nodules were subsequently reviewed using a picture archive and communications system (PACS). These nodules were divided into four sub-groups according to the Bethesda system^6^. For statistical analysis, Bethesda categories III and IV were grouped together, as were Bethesda categories V and VI (Fig. 2).

### Ultrasonography and fine-needle aspiration

Routine ultrasound examinations were performed using a real-time ultrasound scanner with an 8–14 MHz linear transducer (Mylab 90, Esaote Medical System, Italy) by two radiologists (CZ with 13 years’ experience, PTH with 20 years’ experience). FNA was recommended if any of the following ultrasonographic features were present: (a) marked hypoechogenicity (lower echogenicity than the cervical strap muscles), (b) microlobulated shape, (c) irregular margin, (d) microcalcification, (e) greater height than width, and (f) capsular invasion. All ultrasound-guided FNAs were performed by the same radiologist. For each FNA, the patient lay in the supine position with the neck exposed and supported by a pillow, and the head extended. For local anesthesia, 1–2 mL of 1% lidocaine was used for local anesthesia, followed by 5–10 passes with 23-gauge needles (Hakko, Japan) using free-hand capillary aspiration.

Each lesion was aspirated at least twice without suction. The aspirated sample was expelled onto a glass slide and immediately placed in 95% alcohol for papanicolaou staining. The remaining material in the syringe was rinsed in saline and processed for cell block processing. A rapid on-site evaluation by a staff cytopathologist (KX, with 5 years’ experience) was performed in nearly all thyroid FNAs to determine the adequacy of the sample. A cytopathologist (QY) with 10 years of experience made the diagnosis in the pathology department. The cytological results were reported according to the Bethesda System for Reporting Thyroid Cytopathology^6^. This methodology has been applied in our institution since 2011.

Surgery was recommended for patients with nodules that had: (1) suspicious ultrasonographic features; (2) maximal diameter over 4 cm; (3) FNA Bethesda Category ≥III; (4) compression symptoms, and; (5) suspicious cervical lymph nodes. Otherwise, clinical follow-up was suggested, unless surgery was specifically requested by the patient. Surgery was also recommended for patients with nodular enlargement during clinical follow-up.

### Intraoperative frozen section

During surgery, pathologists performed intraoperative FS to confirm benignancy or malignancy in patients who underwent thyroid lobectomy. FS results were categorized as: 1, benign, including nodular goiter, thyroid hyperplasia (Graves’ disease), thyroiditis, lymphocytic/autoimmune thyroiditis (Hashimoto thyroiditis), and granulomatous thyroiditis (De Quervain thyroiditis); 2, malignant, including papillary carcinoma, follicular carcinoma (widely invasive), poorly differentiated (insular) carcinoma, undifferentiated (anaplastic) carcinoma, malignant lymphoma, and medullary carcinoma, or; 3, deferred (indeterminate results).

### Statistical analysis

Statistical analysis was performed using SPSS software (Version 19, IBM, USA). The level of statistical significance was set at *P* < 0.05. The malignancy rate between groups and between FNA and FS were compared. The accuracy rate of FNA and FS based on the final histological results were calculated, respectively. Comparisons of malignancy rate were performed by using the McNemar’s test.
